# Valorization of Xylose-Rich Medium from *Cynara cardunculus* Stalks for Lactic Acid Production via Microbial Fermentation

**DOI:** 10.3390/polym16243577

**Published:** 2024-12-21

**Authors:** Gianfrancesco Russo, Mattia Gelosia, Giacomo Fabbrizi, Mariarosaria Angrisano, Grazia Policastro, Gianluca Cavalaglio

**Affiliations:** 1 CIRIAF, Interuniversity Research Centre on Pollution and Environment “M.Felli”, University of Perugia, Via G. Duranti 67, 06125 Perugia, Italy; gianfrancesco.russo@dottorandi.unipg.it (G.R.); mattia.gelosia@collaboratori.unipg.it (M.G.); giacomo.fabbrizi@unipg.it (G.F.); 2 Department of Engineering, Pegaso Telematic University, 80143 Naples, Italy; mariarosaria.angrisano@unipegaso.it (M.A.); grazia.policastro@unipegaso.it (G.P.)

**Keywords:** lignocellulosic residues, biorefinery crop, lactic acid bacteria, xylose, PLLA, PKA pathway, PP pathway, EMP pathway

## Abstract

Lactic acid (LA) is a versatile, optically active compound with applications across the food, cosmetics, pharmaceutical, and chemical industries, largely driven by its role in producing biodegradable polylactic acid (PLA). Due to its abundance, lignocellulosic biomass is a promising and sustainable resource for LA production, although media derived from these matrices are often rich in xylose and contain growth inhibitors. This study investigates LA production using a xylose-rich medium derived from *Cynara cardunculus L. altilis* DC stalks treated through steam explosion and enzymatic hydrolysis. The lactic acid bacteria strains *Lacticaseibacillus casei*, *Paucilactobacillus vaccinostercus*, and *Pediococcus pentosaceus* were grown on natural media, achieving yields of 0.59, 0.57, and 0.58 g LA/g total carbon consumed, respectively. Remarkably, on xylose-rich media, all supplied sugar was consumed, with LA yields comparable to those on complex media. These findings highlight the adaptability of these strains in the presence of inhibitors and support the potential of lignocellulosic biomass as a low-cost and sustainable substrate for effective PLA production.

## 1. Introduction

Lactic acid (LA), also known as 2-hydroxypropionic acid, is a versatile compound widely utilized across multiple industries, including food, cosmetics, pharmaceuticals, and chemicals [1]. In 2021, the global LA market was valued at approximately $2.9 billion [2], primarily due to its crucial role in the production of polylactic acid (PLA), a biodegradable and compostable polyester [3]. PLA offers a sustainable alternative to petrochemical plastics, contributing significantly to the reduction of carbon emissions [4]. LA can be produced through chemical synthesis or microbial fermentation [5], with over 90% of global production now relying on the latter method [6]. Fermentation allows for the direct production of optically pure D-LA or L-LA, depending on the specific microbial strain used [7], and is a cost-effective and environmentally friendly alternative to traditional chemical processes [8]. Lactic acid bacteria (LAB) generate LA through two main fermentation pathways, homofermentative and heterofermentative, which are determined by the end products of the fermentation process [9]. Obligatory homofermentative LAB convert glucose into pyruvate via the Embden–Meyerhof–Parnas pathway (EMP-P) under anaerobic conditions with an excess of substrate. This pyruvate is then further converted into lactate, yielding two moles of LA for every mole of glucose. Some strains can also metabolize hexoses and pentoses through the pentose phosphate pathway (PP-P), potentially achieving a theoretical maximum yield of 1.67 moles of LA per mole of glucose [10,11]. Heterofermentative LAB can be classified into obligatory and facultative types [12]. Obligatory heterofermentative LAB can ferment both hexoses and pentoses using the phosphoketolase pathway (PK-P). By contrast, facultative heterofermentative LAB metabolize hexoses via the EMP-P and pentoses through the PK-P. Consequently, acetic acid (AA) production in facultative heterofermentative LAB does not occur until all glucose has been consumed, signaling a shift from homofermentative to heterofermentative metabolism. For instance, *Paucilactobacillus vaccinostercus* preferentially metabolizes pentoses and has been isolated from various sources, including cow dung, fermented tea leaves, and cereals [13]. Studies found that *P. vaccinostercus* is a pentose-dependent LAB related to pectin degradation [14]. It was observed that *P. vaccinostercus*, grown in a YP medium with 50 g/L of xylose, produced 20 g/L of LA within 48 h of fermentation [15]. Another LAB capable of utilizing pentoses as a carbon source for fermentation is *Pediococcus pentosaceus*. The growth and LA production (LA_p_) of this microorganism were studied in a complex glucose medium. Optimal growth was achieved in a CO₂ atmosphere at 40 °C and a pH of 6.3. During the exponential growth phase, L-LA was primarily produced [16]. One of the highest LA_p_ recorded for *P. pentosaceus* was achieved using the electrodeionization technique. This approach employed intermittent fed-batch fermentation with partial broth removal, resulting in a final LA_p_ of 184 g/L at the end of the process [17]. Another example of an LAB with this metabolic versatility is *Lacticaseibacillus casei*, which can utilize sugars through the EMP-P and PK-P, as shown by carbon flux balance studies [18]. Using an exponential feeding strategy with a glucose solution (850 g/L) and 1% yeast extract, *L. casei* achieved a LA_p_ of 210 g/L, of which 180 g/L was L-LA [19]. The feasibility of LA production through fermentation is influenced by various factors, particularly the cost of raw material, which play a significant role in determining the economic viability of the process [20]. In order to reduce LA production costs, research is focusing on the use of natural media derived from residual biomass. For example, banana crop residue hydrolysate was used as a culture medium for the growth of *P. pentosaceus* HLV1. After 72 h of fermentation, the strain *P. pentosaceus* HLV1 produced a significant amount of LA (56.5 g/L) [21]. Another natural medium used was soybean stalk hydrolysate, where *L. casei* yielded an LA conversion (Y_LA_) of 56% [22]. Lignocellulosic biomass presents an abundant and sustainable resource for the production of sugars, with over 181.5 billion tons produced annually worldwide [23]. The use of food crops as feedstock for biochemical production could be substantially reduced by exploiting lignocellulosic biomass. This approach is essential for clean and sustainable processes and products with low carbon emissions. In addition to glucose, xylose is a key sugar found in lignocellulosic residues, being the primary hemicellulosic sugar in hardwoods and agricultural by-products and comprising up to 25% w/w of the dry biomass in certain plant species [24]. Its abundance and ease of extraction make xylose an ideal feedstock for producing bulk chemicals. However, the absence of the xylose metabolic pathway in many microbial systems cause xylose valorization to be alienated and abandoned, a major bottleneck in the commercial viability of LCB-based biorefineries [25].

Among lignocellulosic crops, *Cynara cardunculus L. altilis* DC (cardoon), native to the Mediterranean and part of the Asteraceae family, stands out for its minimal water and fertilizer requirements [26]. This perennial herbaceous plant offers significant advantages, including low agricultural input, high biomass productivity (10–20 tons per hectare of dry biomass), and low moisture content [27]. Its resilience on marginal lands and its high holocellulose content (about 50–60%) make cardoon residual stalks an excellent candidate for LA production [28]. However, this lignocellulosic matrix is recalcitrant towards microbial fermentation, making a cost-effective pre-treatment essential to hydrolyze the holocellulose and achieve high carbohydrate yields [29]. One of the most common pretreatment methods is steam explosion, a process in which lignocellulosic biomass is subjected to high-pressure steam at temperatures between 160 and 250 °C for a very short time [30]. This treatment is followed by explosive decompression, which disrupts the rigid structure of the biomass [30]. The high temperature facilitates the hydrolysis of acetyl groups, releasing AA that catalyzes the hydrolysis of polysaccharides, primarily hemicelluloses, in a process known as auto-hydrolysis [31]. The resulting liquid fraction (LF) can be directly fermented while the cellulose-rich pulp (SF) is enzymatically hydrolyzed to produce a glucose-rich hydrolysate (HY). The fermentation of LF can be hindered by the inhibitory effects of certain compounds, such as AA, furfural, and 5-hydroxymethylfurfural (5-HMF), which are released during the pretreatment process [9]. The reduction of inhibitor compounds during the pre-treatment has to be taken into account without decreasing the release of monosaccharides. Cavalaglio et al. [28] optimized steam explosion pretreatment for cardoon stalks, producing a high-quality LF rich in xylose with low levels of inhibitors, along with an easily digestible SF. The cardoon stalks, comprising approximately 40% of the total dry biomass, represent a valuable source of chemicals for industrial applications. Their notably high cellulose content can be hydrolyzed into fermentable sugars [32]. For instance, cardoon HY was used as a microbial growth medium for the production of poly-3-hydroxybutyrate [33]. The study focused on analyzing the pure HY, pure LF, and different HY–LF ratios obtained from the acid-catalyzed steam explosion of cardoon stalks as a potential microbial growth medium alternative to the conventional media available on the market.

To the best of our knowledge, natural microbial media obtained from cardoon stalks have not yet been tested for LA production and could serve as a low-cost growth media, potentially reducing PLA production costs. This study aims to address this gap by employing cardoon HY and LF as microbial media for various LAB strains. The analysis was conducted through the screening of three microbial strains *Lacticaseibacillus casei* DSM 20011, *Paucilactobacillus vaccinostercus* DSM 20634, and *Pediococcus pentosaceus* DSM 20333, capable of utilizing xylose as a carbon source and growing in media containing inhibitors.

## 2. Materials and Methods

### 2.1. Pretreatment of Cardoon

Cardoon (*Cynara cardunculus L. altilis* DC) was supplied by Matrica S.p.A. (Porto Torres, Italy). The raw material, consisting of stalks, was cut into 2–3 cm pieces using a woodchipper. The woodchips, with a moisture content of 6.87% (*w*/*w*), were ground into a powder with an average particle size of 0.5 mm using a laboratory rotary blade mill (RETSCH, Haan, Germany). The powder was characterized using the analytical methods for biomass outlined by the National Renewable Energy Laboratory (NREL, Golden, CO, USA) [34]. All characterizations were performed in triplicate, and the results are shown in Table 1.

The wood chips underwent steam explosion pre-treatment using the condition established by Cavalaglio et al. [28] (166 °C, 1.45% (*w*/*w*) H_2_SO_4_, and 10 min) to minimize the production of the inhibitors. At the end of the pretreatment, two fractions were obtained: SF rich in cellulose and lignin, and an LF rich in pentose sugars, mainly xylose, mannose, galactose, and arabinose, derived from the acid hydrolysis of hemicellulose. As stated by Cavalaglio et al. [28], xylose is the main monosaccharide released from the hydrolysis of cardoon hemicellulose. The LF also contained inhibitors, such as AA, furfural, and 5-HMF, formed due to the partial degradation of sugars under high temperatures and acidic conditions. The SF was washed with deionized water, and the subsequent enzymatic hydrolysis was carried out in deionized water using cellulolytic enzymes Cellic^®^ CTec2 (Merck, Darmstadt, Germany), with a solid loading of 10% (*w*/*w*) and an enzyme dosage of 60 FPU/g cellulose, to obtain a HY rich in glucose [35]. A compositional analysis was conducted on both HY and LF, with the results summarized in Table 2.

### 2.2. Bacterial Strain and Growth Conditions

The LAB investigated in this work were: *L. casei* DSM 20011, *P. vaccinostercus* DSM 20634, and *P. pentosaceus* DSM 20333, all obtained from the Deutsche Sammlung von Mikroorganismen und Zellkulturen GmbH (DSMZ, Leibniz, Germany). After reactivating following the supplier instruction, the stock cultures were maintained at −80 °C in the pre-culture MRS (DeMan–Rogosa–Sharpe) broth media (Merck, Darmstadt, Germany) with 20% (*v*/*v*) glycerol. The maintenance of the strains was performed on MRS agar with periodic transfer. The composition of MRS was as follows: 2 g/L K_2_HPO_4_, 20 g/L glucose, 0.2 g/L MgSO_4_·7H_2_O, 0.05 g/L MnSO_4_·4H_2_O, 8 g/L meat extract, 10 g/L peptone, 5 g/L CH_3_COONa·3H_2_O, 2 g/L triammonium citrate, 4 g/L yeast extract, and 1 mL/L Tween 80. The media were sterilized by an autoclave at 121 °C for 15 min and the pH of the culture media was adjusted to 6.7 with 2 M NaOH.

### 2.3. Preliminary Analysis

Analyses of the maximum Y_LA_ from the LAB were performed during the preliminary phase using two complex media, which were similar to MRS broth, with the exception of the carbon source. Medium A contained glucose (20 g/L), while medium B contained xylose (20 g/L). After sterilization in an autoclave at 121 °C for 15 min, the sugars underwent partial thermal degradation, resulting in final concentrations of 16 g/L for glucose and 10 g/L for xylose. Notably, the xylose in medium B experienced greater degradation, generating compounds that contributed to a drop in the medium’s pH. The pH was subsequently adjusted to 6.7 by adding 2 M NaOH. The pre-inoculum was prepared by transferring a loopful of 48-h-old cells, grown on MRS agar, into 50 mL of pre-culture medium (with the same composition of media A and B). After 24 h of incubation at 30 °C with continuous shaking at 180 rpm (KS 4000i control, IKA®, Staufen, Germany), the inoculum concentration was determined by measuring the optical density at 600 nm (OD_600_). An aliquot of the pre-culture medium was inoculated into a 100 mL orbital shaker flasks containing 50 mL of each complex medium to achieve an initial OD_600_ of 0.1. The cultures were then incubated for 96 h at 30 °C with continuous shaking at 180 rpm. The fermentation time in the preliminary analysis was set to 96 h to maintain uniformity with LA production tests.

### 2.4. Fermentation for LA Production

Pure HY, HY–LF mixtures, and pure LF were used as culture media for LAB growth and LA production. The chemical composition of each medium is shown in Table 3. To avoid excessive sugars degradation, the resulting increase in inhibitors, and to prevent contamination, before adding the inoculum, the media underwent a tyndallization process. An incubation at pH 7 and 30 °C for 24 h was performed to allow spores to germinate between each heat treatment (80 °C for 30 min). At the end of the final step of tyndallization, the pH of the different natural media was adjusted to 6.7 by adding 2 M NaOH. An amount of 50 g/L of calcium carbonate was added to the medium to maintain the pH relatively stable. The pre-inoculum was prepared by using 50% of medium A or medium B and 50% of the culture media prepared using HY and LF. Due to media with stressful conditions, the lag phase could last longer, so the fermentation was stopped after 96 h to ensure the complete depletion of carbohydrates.

### 2.5. Analytical Procedures

The cultures were centrifuged at 8000× *g* for 5 min and the supernatants were diluted to the desired extent with deionized water. LA was solubilized by adding 1 M H_2_SO_4_ solution. A HPLC analysis was performed to determine the concentration of monosaccharides, LA, 5-HMF, furfural, and AA. The HPLC system used was a Dionex UltiMate 3000 (Thermo Fisher Scientific, Sunnyvale, CA, USA), equipped with a Bio-Rad Aminex HPX-87H column (Bio-Rad, CA, USA) and an index detector (RefractoMax 521, Thermo Fisher Scientific, Sunnyvale, CA, USA). The flow rate was set at 0.6 mL/min, with a column temperature of 50 °C. The mobile phase consisted of 5 mM H_2_SO_4_ in aqueous solution. The peaks for xylose, mannose, and galactose co-eluted, forming a single peak referred to as XMG. The LA_p_ by the different microorganisms enabled the calculation of the Y_LA_ Equation (1).

Y_LA_ = (LA_p_)/(TC_c_) (1)

where TC_c_ is the total carbon consumed (g/L), consisting of glucose, XMG (xylose, mannose, galactose), and arabinose contained in the different natural media investigated.

### 2.6. Enantiomeric Purity

To assess the enantiomeric purity of LA in the post-fermentation broth, the NZYtech d-/l-Lactic Acid Kit (NZYtech, Lisbon, Portugal) was employed. Analyses were conducted on aliquots of centrifuged samples for acid quantification. The tests, including dilution steps, were performed in triplicate, and the results were subsequently averaged. L-LA concentrations were calculated by applying the L/D fractions to the total acid concentration, which was determined via HPLC.

### 2.7. Statistical Analysis

Statistical analysis was conducted using RStudio (Posit PBC, Boston, MA, USA) a freely accessible integrated environment for statistical computing and data visualization. The Y_LA_ was compared among the different LAB analyzed and the various culture media used, employing two-way ANOVA and the Tukey–Kramer test. The assumptions of homogeneity of variance and normality of distribution were validated using the Levene’s test and the Shapiro–Wilk test.

## 3. Results and Discussion

**Figure 1:**
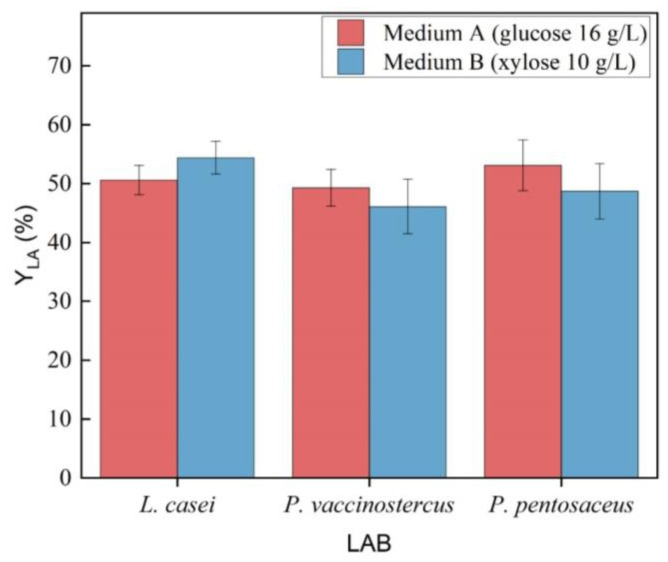
Analysis of Y_LA_ by the three microbial strains using the natural media, medium A with glucose (16 g/L), and medium B with xylose (10 g/L).

During the preliminary phase of this study, the maximum Y_LA_ produced by the LAB was evaluated, using glucose and xylose as the starting sugars (Figure 1). In Medium A, all three microbial strains consumed the available glucose, with *P. pentosaceus* reaching a maximum Y_LA_ of 0.53 g LA_p_ /g TC_c_, given a theoretical maximum Y_LA_ of 1.0 g LA_p_/g TC_c_. The Y_LA_ among the three strains showed no statistically significant differences (*p*-value = 0.132), and by the end of fermentation, the pH stabilized around 4. Interestingly, all microbial strains fully consumed the available xylose in medium B (10 g/L), with no statistically significant differences in Y_LA_ compared to medium A (*p*-value = 0.297). The highest Y_LA_ was observed for *L. casei* (0.54 g LA_p_/g TC_c_), which was not significantly higher than that of *P. vaccinostercus* (0.46 g LA_p_/g TC_c_) and *P. pentosaceus* (0.48 g LA_p_/g TC_c_). All three Y_LA_, however, were found to be very close to the theoretical maximum yield of xylose (0.6 g LA_p_/g TC_c_). All the LAB produced low concentrations of AA during the fermentation, which could be explained by the use of the PK-P, in accordance with the literature that describes the three strains as heterofermentative [12].

To assess the impact of natural media derived from the thermo-physical pretreatment of cardoon on LA fermentation, the three LAB strains were cultured in batch fermentation using various HY and LF mixtures (Table 3). As shown in Table 4, increasing the LF portion led to higher AA and xylose concentrations in the media, reaching its maximum at 100% LF. A slight decrease of 5-HMF and furfural was observed due to thermal degradation in other unanalyzed inhibitor compounds [28,36].

Surprisingly, despite the stressful conditions, all the microbial strains survived in all the tested media, as demonstrated by sugar consumption and LA_p_ (Table 4). It is possible that the investigated strains had a natural attitude to endure an acidic environment, due to a change in membrane composition. The change in membrane in composition resulted in a reduced permeability to organic acids which is probably extended to other inhibitory compounds, such as 5-HMF and furfural [37]. In standard conditions, both inhibitors diffuse through the microbial membrane and have detrimental effects on cell functioning [38]. This hypothesis is corroborated by the fact that the 5-HMF and furfural concentration at the end of fermentation is almost equal to the initial one, while AA concentration increases (Table 4). For cost efficient conversion of solubilized sugars present in the LF, it is desirable to have microbial strains which can maintain optimum metabolic activity in the presence of the inhibitors. Notably, the supplied sugars were almost totally consumed by LAB strains in all natural media (Table 4), demonstrating their ability to use xylose and grow in the presence of stressors. The chance to exploit media obtained by using LF as a carbon source is very interesting despite its relatively low sugar content. The LF is currently underused or discharged inside the lignocellulosic biorefinery due to the high cost of its detoxification [38,39], so its direct use in the fermentation process is a value-added option. Furthermore, no ethanol production was observed, reducing the possible suspect of contamination mediated by ethanol producing microorganisms.

**Figure 2:**
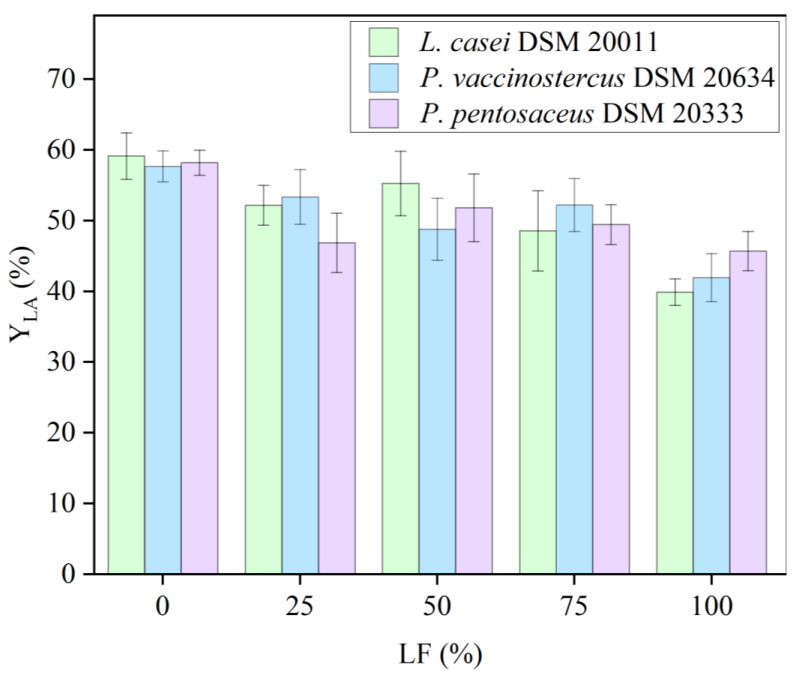
Evaluation of the Y_LA_ of the three LAB strains examined, *L. casei*, *P. vaccinostercus*, and *P. pentosaceus*, with increasing LF concentrations in the culture media.

The statistical analysis revealed that the three LAB strains analyzed did not show significant differences among them in all the culture media investigated (*p*-value = 0.82). In terms of Y_LA_ (Figure 2), the pure HY medium performed better than the other media due to the high concentration of glucose, which plays a crucial role in the fermentative LA_p_.

Due to lower initial carbon content, the LA_p_ decreased at increasing LF content, while the Y_LA_ was unchanged except for the pure LF (*p*-value = 0.0001). A slightly higher decrease in LA_p_ was observed using the pure LF. This can be attributed to the different conversion yields of xylose compared to glucose, which is lower due to the production of not only LA but AA, as well as the potential presence of different inhibitors originated by 5-HMF and furfural degradation [36].

Despite this, it is noteworthy that no statistically significant differences in Y_LA_ were observed among the use of 25% LF, 50% LF, and 75% LF in all three microorganisms (*p*-value = 0.91, *p*-value = 0.99, and *p*-value = 0.98, respectively). Therefore, up to 75% LF can be used in the culture medium, utilizing a fraction derived from the steam explosion process that is rich in pentoses and still considered a by-product. Furthermore, the Y_LA_ values were slightly higher compared to those achieved with complex media (media A and B), highlighting the potential of low-cost, natural microbial substrates for efficient LA production. Full results of the two-way ANOVA and the Tukey–Kramer test are shown in the Supplementary Materials.

The enantiomeric analysis revealed the production of L-LA with high optical purity, exceeding 90%, in all three LAB strains studied, with no significant variations in purity observed across different HY–LF ratios for each microorganism. The production of optically pure L-LA enables the synthesis of PLLA. Compared to PDLA (produced through the polymerization of D-lactide), PLLA exhibits higher crystallinity, chemical stability, and greater resistance to enzymatic degradation [40,41]. Additionally, the degradation of PLLA generates L-LA, which is safe for the human body, while D-LA from PDLA poses slight toxicity risks [42].

The best results, in terms of LA_p_ and Y_LA_ for the three microorganisms analyzed, *L. casei*, *P. vaccinostercus*, and *P. pentosaceus*, were achieved using pure HY as the culture medium, in line with findings reported in the literature with other feedstocks (Table 5). Future research will be focus on evaluating LA_p_ and Y_LA_ by increasing the starting carbohydrate load through the use of more concentrated HY, as well as analyzing the fermentation kinetics of these LAB.

## 4. Conclusions

This study demonstrates the potential of three LAB strains (*L. casei*, *P. vaccinostercus*, and *P. pentosaceus*) to effectively produce LA from complex media and natural media obtained by treating *Cynara cardunculus L. altilis* DC stalks. The strains showed a similar Y_LA_ across different sugar sources, whether using glucose or xylose, with no significant yield differences among the strains. Even in media with high inhibitor levels, all the strains maintained LA_p_ and Y_LA_. The findings highlight the adaptability of these LAB strains to environmental stresses, supporting their use in pentose-rich media derived from lignocellulosic biomass, which could help lower bioprocessing costs. The enantiomeric analysis revealed the production of L-LA with high optical purity (exceeding 90%), enabling the synthesis of PLLA. These microbial strains offer a promising solution for LA production using underutilized lignocellulosic substrates, potentially lowering the PLA production costs associated with feedstock supply. Future research will focus on using concentrated natural media added with only a nitrogen source and reducing fermentation time to increase the productivity of LA fermentation.

## Figures and Tables

**Table 1:** Chemical composition of cardoon stalks.

Cellulose	Hemicellulose	Acetyl Group	Pectin	Lignin *	Extractives	Ash
33.89 ± 0.12	19.23 ± 0.05	4.78 ± 0.15	4.26 ± 0.42	18.49 ± 1.87	7.63 ± 0.27	6.81 ± 0.18

* Lignin as acid insoluble lignin.

**Table 2:** Chemical compounds in HY and LF.

	Concentration (g/L)					
	Glucose	XMG ^1^	Arabinose	AA	5-HMF	Furfural
LF	2.7	23.3	0.6	3.1	0.12	0.85
HY	45.4	5.4	-	0.2	-	-

^1^ xylose, mannose, galactose.

**Table 3:** Chemical composition of Pure HY, HY–LF mixtures, and pure LF.

Culture Medium		Concentration (g/L)				
	Glucose	XMG ^1^	Total Carbon Content	AA	5-HMF	Furfural
Pure HY	45.4	5.4	50.8	0.2	-	-
75HY:25LF	36.41	11.23	47.64	0.9	0.03	0.23
50HY:50LF	25.3	16.78	42.08	1.7	0.05	0.43
25HY:75LF	14.35	20.18	34.53	2.4	0.08	0.61
Pure LF	2.7	23.3	26	3.1	0.12	0.85

^1^ xylose, mannose, and galactose.

**Table 4:** Chemical composition of pure HY, pure LF and different HY–LF ratios media before and after fermentation.

**Pure HY**												
**Strain**	**G_i_ ^a^**	**G_f_ ^b^**	**XMG_i_ ^c^**	**XMG_f_ ^d^**	**LA_i_ ^e^**	**LA_f_ ^f^**	**AA_i_ ^g^**	**AA_f_ ^h^**	**5-HMF_i_ ^i^**	**5-HMF_f_ ^l^**	**F_i_ ^m^**	**F_f_ ^n^**
DSM 20011 ^o^	39.67± 2.56	2.16 ± 1.08	3.28 ± 1.18	-	-	25.12 ± 1.15	0.49 ± 0.21	0.54 ± 0.16	-	-	-	-
DSM 20634 ^p^	39.67± 2.56	1.96 ± 1.55	3.28 ± 1.18	-	-	23.36 ± 1.88	0.49 ± 0.21	0.68 ± 0.24	-	-	-	-
DSM 20333 ^q^	39.67± 2.56	1.87± 1.26	3.28 ± 1.18	-	-	26.08 ± 0.87	0.49 ± 0.21	0.71 ± 0.31	-	-	-	-
**75HY:25LF**												
**Strain**	**G_i_**	**G_f_**	**XMG_i_**	**XMG_f_**	**LA_i_**	**LA_f_**	**AA_i_**	**AA_f_**	**5-HMF_i_**	**5-HMF_f_**	**F_i_**	**F_f_**
DSM 20011	33.41	0.85± 0.41	8.23	-	-	22.73 ± 2.15	3.54 ± 1.07	3.82 ± 1.16	0.31 ± 0.05	0.33 ± 0.06	0.32 ±0.06	0.32 ± 0.05
DSM 20634	33.41	1.22 ± 0.79	8.23	-	-	23.13 ± 1.78	3.54 ± 1.07	3.88 ± 0.43	0.31 ± 0.05	0.34 ± 0.09	0.32 ±0.06	0.28 ± 0.03
DSM 20333	33.41	1.54 ± 1.12	8.23	-	-	19.47 ± 1.76	3.54 ± 1.07	3.91 ± 0.74	0.31 ± 0.05	0.36 ± 0.10	0.32 ±0.06	0.31 ± 0.07
**50HY:50LF**												
**Strain**	**G_i_**	**G_f_**	**XMG_i_**	**XMG_f_**	**LA_i_**	**LA_f_**	**AA_i_**	**AA_f_**	**5-HMF_i_**	**5-HMF_f_**	**F_i_**	**F_f_**
DSM 20011	18.3571 ± 2.15	-	13.48 ± 1.27	-	-	19.28± 2.09	3.98 ± 1.1	4.14 ± 1.03	0.27 ± 0.03	0.31 ± 0.07	0.29 ± 0.03	0.29 ± 0.06
DSM 20634	18.3571 ± 2.15	-	13.48 ± 1.27	-	-	16.74 ± 3.19	3.98 ± 1.1	4.60 ± 0.38	0.27 ± 0.03	0.30 ± 0.07	0.29 ± 0.03	0.25 ± 0.05
DSM 20333	18.3571 ± 2.15	-	13.48 ± 1.27	-	-	18.02 ± 3.79	3.98 ± 1.1	4.11 ± 0.79	0.27 ± 0.03	0.30 ± 0.07	0.29 ± 0.03	0.27 ± 0.04
**25HY:75LF**												
**Strain**	**G_i_**	**G_f_**	**XMG_i_**	**XMG_f_**	**LA_i_**	**LA_f_**	**AA_i_**	**AA_f_**	**5-HMF_i_**	**5-HMF_f_**	**F_i_**	**F_f_**
DSM 20011	11.75± 2.31	-	15.41 ± 0.29	3.26 ± 0.68	-	13.73 ± 1.09	4.13 ± 0.40	4.17 ± 0.60	0.24 ± 0.03	0.22 ± 0.08	0.23 ± 0.02	0.22 ± 0.06
DSM 20634	11.75± 2.31	-	15.41 ± 0.29	2.85 ± 0.54	-	14.32 ± 1.67	4.13 ± 0.40	4.12 ± 0.22	0.24 ± 0.03	0.25 ± 0.07	0.23 ± 0.02	0.23 ± 0.03
DSM 20333	11.75± 2.31	-	15.41 ± 0.29	2.68 ± 0.61	-	12.98 ± 1.23	4.13 ± 0.40	4.24 ± 0.35	0.24 ± 0.03	0.36 ± 0.19	0.23 ± 0.02	0.23 ± 0.05
**Pure Lf**												
**Strain**	**G_i_**	**G_f_**	**XMG_i_**	**XMG_f_**	**LA_i_**	**LA_f_**	**AA_i_**	**AA_f_**	**5-HMF_i_**	**5-HMF_f_**	**F_i_**	**F_f_**
DSM 20011	2.41 ± 0.38	-	16.22 ± 0.39	2.59 ± 1.76	-	7.12 ± 0.35	4.82 ± 0.60	4.97 ± 0.84	0.19 ± 0.01	0.19 ± 0.03	0.26 ± 0.01	0.22 ± 0.03
DSM 20634	2.41 ± 0.38	-	16.22 ± 0.39	2.28 ± 1.93	-	7.68 ± 0.39	4.82 ± 0.60	5.18 ± 0.70	0.19 ± 0.01	0.22 ± 0.01	0.26 ± 0.01	0.20 ± 0.02
DSM 20333	2.41 ± 0.38	-	16.22 ± 0.39	3.59 ± 1.46	-	7.41 ± 0.88	4.82 ± 0.60	5.34 ± 1.26	0.19 ± 0.01	0.21 ± 0.01	0.26 ± 0.01	0.21 ± 0.02

^a^ Initial glucose content; ^b^ final glucose content; ^c^ initial xylose, mannose, and galactose content; ^d^ final xylose, mannose, and galactose content; ^e^ initial acetic acid content; ^f^ final acetic acid content; ^g^ initial lactic acid content; ^h^ final lactic acid content; ^i^ initial 5-HMF content; ^l^ final 5-HMF content; ^m^ initial furfural content; ^n^ final furfural content; ^o^ *L. casei*; ^p^ *P. vaccinostercus*, ^q^ *P. pentosaceus*.

**Table 5:** Summary of LA_p_ and Y_LA_ achieved using different feedstock as culture media and different microbial strains.

Microorganism	Feedstock	LA_p_	Y_LA_	References
*Lb. casei* DSM 20011	Cardoon stalks hydrolysate	25.12	0.59	**This study**
*P. vaccinostercus* DSM 20634	Cardoon stalks hydrolysate	23.36	0.57	**This study**
*P. pentosaceus* DSM 20333	Cardoon stalks hydrolysate	26.08	0.58	**This study**
*Lb. casei*	Banana wastes	-	0.10	[43]
*Lb. casei* ATCC 10863	Ram horn hydrolysate	44	0.44	[44]
*Lb.casei*	Sugarcane bagasse	21.3	0.63	[45]
*P. pentosaceus* KTU05-8	Hydrolyzed cheese whey	21.45	0.396	[45]
*P. pentosaceus* KTU05-9	Hydrolyzed cheese whey	25.49	0.519	[45]
*P. pentosaceus* KTU05-10	Hydrolyzed cheese whey	19.46	0.396	[46]
*L. vaccinostercus*	YP medium with 50 g/L of xylose	20	-	[46]
*L. vaccinostercus*	MRS medium with 50 g/L of xylose	26	-	[47]

## Data Availability

Data available on request. The original contributions presented in the study are included in the article/Supplementary Material, further inquiries can be directed to the corresponding authors. The data presented in this study are available on request from the corresponding author. The data are not publicly available due to [insert reason here].

## Supplementary Materials

The following supporting information can be downloaded at: https://www.mdpi.com/article/10.3390/polym16243577/s1, Table S1: Validating the assumption of normal sample distribution and homogeneity of variance of Y_LA_ using complex medium; Table S2: Analysis of the effects of complex medium A and B on the Y_LA_; Table S3: Validating the assumption of normal sample distribution and homogeneity of variance of Y_LA_; Table S4: Analysis of the effects of different microorganisms and LF:HY ratios on the YLA; Table S5: Evaluation of Y_LA_ through multiple comparisons between different groups treated with various LF:HY ratios.
