# Supplementary material for: Valorization of Xylose-Rich Medium from Cynara cardunculus Stalks for Lactic Acid Production via Microbial Fermentation
Source: Polymers (Basel). 2024 Dec 21;16(24):3577. doi: 10.3390/polym16243577 (PMC11679648; doi:10.3390/polym16243577)
Supplement: Supplementary file 1 [file polymers-16-03577-s001.zip › polymers-3331323-supplementary.pdf]

Table S1. Validating the assumption of normal sample distribution and homogeneity of variance of Y_{LA} using complex medium.

Levene test (H_0: Homogeneity of variance)	p-value [1]
	0.6325
**Shapiro test (H_0: Normal distribution)**	**p-value [2]**
	0.7996

[1] null hypothesis (H_0) was rejected for p-value<0.05.

[2] null hypothesis (H_0) was rejected for p-value<0.05.

Table S2. Analysis of the effects of complex medium A and B on the Y_{LA}.

Two way ANOVA	
**Microorganism**	**p-value [3]**
	0.132
**Complex medium**	**p-value [4]**
	0.297

[3] null hypothesis (H_0) was rejected for p-value<0.05.

[4] null hypothesis (H_0) was rejected for p-value<0.05.

Table S3. Validating the assumption of normal sample distribution and homogeneity of variance of Y_{LA}.

Levene test (H_0: Homogeneity of variance)	p-value [5]
	0.9554
**Shapiro test (H_0: Normal distribution)**	**p-value [6]**
	0.1877

[5] null hypothesis (H_0) was rejected for p-value<0.05.

[6] null hypothesis (H_0) was rejected for p-value<0.05.

Table S4. Analysis of the effects of different microorganisms and LF:HY ratios on the Y_{LA}.

Two way ANOVA	
**Microorganism**	**p-value [7]**
	0.818
**Culture medium**	**p-value [8]**
	2.23×10^{-6}

[7] null hypothesis (H_0) was rejected for p-value<0.05.

[8] null hypothesis (H_0) was rejected for p-value<0.05.

Table S5. Evaluation of Y_{LA} through multiple comparisons between different groups treated with various LF:HY ratios.

Tukey Test

**Culture medium**	**p-value [9]**
75HY:25LF vs. Pure HY	0.0005182
50HY:50LF vs. Pure HY	0.0013190
25HY:75LF vs. Pure HY	0.0000995
Pure LF vs. Pure HY	0
50HY:50LF vs. 75HY:LF25	0.9977934
75HY:25LF vs. 25HY:75LF	0.9824728
Pure LF vs. 75HY:25LF	0.0000225
25HY:75LF vs. 50HY:50LF	0.9114973
Pure LF vs. 50HY:50LF	0.0000084
Pure LF vs. 25HY:75LF	0.0001209

[9] null hypothesis (H_0) was rejected for p-value<0.05.
